# Mindfulness for pain, depression, anxiety, and quality of life in people with spinal cord injury: a systematic review

**DOI:** 10.1186/s12883-020-1619-5

**Published:** 2020-01-21

**Authors:** Jasmine Heath Hearn, Ainslea Cross

**Affiliations:** 1 Department of Psychology, Manchester Metropolitan University, 53 Bonsall Street, Manchester, M15 6GX UK; 2 Department of Psychology, University of Derby, Kedleston Road, Derby, DE22 1GB UK

**Keywords:** Meditation, Mind-body, Yoga, Paraplegia, Acceptance

## Abstract

**Background:**

Populations with reduced sensory and motor function, such as spinal cord injury (SCI) are at increased risk of depression, anxiety, pain, and poorer quality of life (QoL). Mindfulness-Based Interventions (MBIs) have been developed with the aim of improving outcomes for people with SCI. To understand the value of MBIs, a systematic review was conducted pertaining to the use of MBIs, and interventions including elements of mindfulness, with people with SCI.

**Methods:**

Databases were reviewed from 1996 to October 2018 (updated January 2020). Eligibility criteria included the assessment of at least one of the common secondary consequences of SCI (i.e. risk of depression, anxiety, pain, and QoL), describe the use of mindfulness training as a component part of an intervention, or as the whole intervention. The Cochrane Collaboration Risk of Bias and The Effective Public Health Practice Project Quality Assessment Tools were utilised for quality appraisals. Two assessors appraised the studies and demonstrated good agreement (Cohen’s *k* = .848*, p* < .001).

**Results:**

Five papers met the inclusion criteria, and demonstrated a range of results of interventions delivered individually, in a group format, in person, and online. Only one study reported significant reductions in pain-related outcomes (with moderate effect sizes), with the remaining studies *(n* = 4*)* demonstrating no change. Four studies described reductions in depressive symptoms and three reported reductions in anxiety. Despite the importance of good QoL as a goal for people with SCI, few studies (*n* = 2) assessed this as an outcome with no improvements reported. Study quality ranged from high to low/weak.

**Conclusions:**

The findings in this review provide mixed support for the use of mindfulness to improve outcomes after SCI. In particular, findings indicate that mindfulness may be particularly effective for improving symptoms of depression and anxiety. This review highlights the requirement for more rigorous, high-quality research, particularly larger randomised-controlled trials with long-term follow-up, in this area. The small number of studies included in the present review mean that conclusions drawn are preliminary and thus reflects the paucity of the research in the area to date.

## Background

Spinal cord injury (SCI) occurs when the spinal cord is damaged (such as through traumatic injury), and often leads to partial or complete loss of motor and/or sensory function below the level of injury [1]. People with SCI may experience many potential secondary physical and psychological consequences, including an increased risk of depression and anxiety (experienced in around 22.2% of the population [2]) and reduced quality of life (QoL [3]). Further, evidence suggests that a complex relationship between depression and chronic pain exists, with each amplifying the other in this population [4]. The wide range of secondary physical and psychological implications of SCI inevitably complicate its management and psychological interventions may play an important role in helping people with SCI to manage such complexities following injury. Indeed, qualitative evidence suggests that people with SCI want better access to psychological interventions [5], making the availability of effective interventions a key priority for the care of people with SCI.

Conflict is evident in the literature on psychological interventions for this population. Some trials examining cognitive behavioural therapy (CBT) have demonstrated a positive impact on depression [4], whilst others have demonstrated no change [6]. A recent systematic review [7] suggests that further evidence is required to evaluate the efficacy of non-pharmacological interventions on a range of psychological outcomes for people with SCI, including depression, anxiety, and quality of life. However, this review focused on a range of mostly physical interventions, including electrical brain stimulation, exercise, acupuncture, hypnosis, transcutaneous electrical nerve stimulation (TENS) and included only one trial of CBT. Further systematic review evidence has evaluated psychosocial interventions for inpatients with SCI, concluding that more work is required to evidence such interventions for this group [8]. Whilst the efficacy of CBT has been systematically reviewed [9, 10], the rapidly growing research interest and evidence surrounding mindfulness-based interventions has not been reviewed systematically, despite the fact that such a review could provide valuable guidance on the future study and application of mindfulness for people with SCI.

In contrast to CBT interventions, which aim to challenge core beliefs and change maladaptive behaviours, acceptance and mindfulness-based interventions (MBIs) aim to facilitate present-moment awareness and acceptance, rather than explicitly trying to change psychological and behavioural responses [11]. Attending to internal experiences (such as physical sensations, thoughts, and emotions) non-judgementally is argued to enhance present-moment awareness [11], leading to cognitive defusion (i.e. reduced identification with thoughts), improved emotional self-regulation and behavioural flexibility [12], and reduced negative emotional reactivity [13]. In populations with neurological damage, such as multiple sclerosis, MBIs have demonstrated benefits in QoL [14], as well as improvements for people experiencing depression [15]. Similarly, MBIs have contributed towards reductions in anxiety and disability in people with chronic back pain [16].

Work is beginning to emerge exploring the role for mindfulness in improving pain and psychosocial outcomes following spinal cord injury. For example, cross-sectional work has demonstrated positive associations between mindfulness and mood in people with SCI [17]. However, it remains difficult to draw conclusions and make evidence-based decisions on the relevance and efficacy of MBIs for people experiencing chronic pain, depression, anxiety, or reduced QoL following SCI.

The aims of this systematic review, therefore, were:

- To synthesise and critically appraise available quantitative and qualitative evidence on the effects of MBIs on pain and pain-related outcomes, depression, anxiety, and QoL in people with SCI.
- To make specific recommendations for future research based on current knowledge.

## Methods

### Eligibility criteria

#### Types of studies

Due to the relative dearth of evidence in this area, all study designs (including randomised, and non-randomised studies) were included in the review to capture the evidence that is available.

#### Participants

Studies including adult participants living with SCI were included, regardless of SCI aetiology. Studies including participants with other conditions were included if results from the SCI subgroup were presented separately from the other groups.

#### Interventions

Interventions describing the use of mindfulness training as a component part of an intervention, or as the whole intervention were included in the review. In addition to specific MBIs, other eligible interventions included meditation, yoga, mindful movement, mindfulness in daily life exercises, and breathing techniques. Comparisons of MBIs with alternative interventions were not required for inclusion in the review, due to the dearth of evidence available.

#### Outcomes

Studies quantitatively evaluating the impact of MBIs on common physical and psychological secondary consequences of SCI were included (with outcomes measured pre- and post- in intervention studies). Where included, follow-up effects were also assessed. These outcomes included at least one of: chronic pain and pain-related outcomes (on any measure of pain intensity and pain relief, such as numerical rating scales and other pain-related outcomes through the use of questionnaires), depression, anxiety, and QoL. Where reported, data on adverse events were also extracted.

#### Further inclusion & exclusion criteria

Further criteria for inclusion were: Published between 1996 and 2019; and published in English in a peer-reviewed journal. Where reported, qualitative comments were also examined.

### Search strategy

#### Data sources

In October 2018 (search updated January 2020), PsycINFO, PsycARTICLES, and MEDLINE were searched using the following search strategy: ((spinal cord injur*) OR Paraplegi* OR Tetraplegi* OR *) AND (Mindful* OR MBI OR Acceptance OR (mind body) OR Meditat*) AND (Pain OR Depress* OR Anxiety OR (Quality of life) OR QoL OR (Mental health)). In addition, reference lists of included papers were screened for additional eligible papers.

#### Data extraction and management

The following pieces of information were extracted from the included studies by the first author (verified by the second author), and are presented in Table 1:

- Year of publication
- Sample size and demographic details of participants (age, gender, aetiology of SCI, ethnicity, time since injury, inpatient vs. outpatient status)
- Research design
- Theoretical framework used (where reported)
- Details of interventions and duration (including control/comparator interventions where included)
- Number of withdrawals and reasons for withdrawal
- Measurement of treatment effect, including *p* values, effect sizes, and confidence intervals (where available) for all outcome measures

**Table 1:** Included study data extraction

Study	N	Sample Characteristics	Research Design	Theoretical Framework	Intervention Modality/ Length and Comparator	Mindfulness Components	Outcome Measures	Number of Withdrawals/ Dropouts & Reasons	Results (*p* values, effect sizes, and 95% CIs where available)
Curtis et al. (2015)	11	Age (M & SD): 48.4 (15) Gender: 90.9% female Ethnicity: not reported Time since injury: M = 157.4 months Etiology of injury: six traumatic injuries, three non-traumatic, two not reported Status: five inpatients, six community-dwelling	Pretest-posttest of an eight-week modified yoga programme	Not reported	Modified eight-week yoga programme: one 45–60 min class per week. No comparator.	Brief (5–10 min) mindfulness meditations focusing on breathing and present moment awareness per class	-Yoga Satisfaction Scale -Toronto Mindfulness Scale -Brief Pain Inventory Short-Form -Pain Catastrophising Scale -Fatigue Severity Scale -Positive and Negative Affect Scale -Cognitive and Affective Mindfulness Scale	6, did not complete course.	No significant changes in depression, pain and pain interference, pain catastrophising, or mindfulness (*p* > .05)
Curtis et al. (2017)	23	Age (M & SD): Yoga group 47.9 (19.51), control group 54.8 (10.11) Gender: not reported Ethnicity: Yoga group 30% Caucasian, 40% south Asian, 10% African-Canadian, 10% east Asian, 10% hispanic. Control group 83.3% Caucasian, 8.3% east Asian, 8.3% other. Time since injury: not reported Etiology of injury: Yoga group seven traumatic injuries, three non-traumatic. Control group 8 traumatic, four non-traumatic Status: not reported	Randomised controlled trial	Not reported	Yoga: Two 50- to 60-min classes per week (approx. 12 h total) for six weeks. Control: wait-list control.	10–15 min mindfulness meditations (breath awareness) per class	-Acceptance and Action Questionnaire -Hospital Anxiety and Depression Scale -General Self-Efficacy Scale -Post-Traumatic Growth Inventory -Connor-Davidson Resilience Scale -Self-Compassion Scale -Five Facet Mindfulness Questionnaire -Brief Pain Inventory Short Form -Pain Catastrophising Scale	5, withdrew for logistical or non-study-related and illness reasons (e.g., unable to provide a doctor’s note to confirm eligibility, moved away, vertigo). 10 of 11 participants completed the yoga intervention. 8 of 12 control participants completed study.	Intervention significantly reduced depression severity (*p* < 0.05) and improved FFMQ-SF – total scores (*p* = 0.09), FFMQ-SF – observing scores (*p* = 0.06), and FFMQ-SF – non-reactivity scores (*p* = 0.04). No significant improvements seen in anxiety, pain and pain catastrophising (*p* >. 05).
Flores et al. (2018)	2	Age (M & SD): Participant 1 39 years, participant 2 31 years Gender: 100% male Ethnicity: not reported Time since injury: two weeks Etiology of injury: Both traumatic Status: both inpatients	Case study	Not reported	Virtual reality enhanced Dialectical Behavioral Therapy Mindfulness Skills Training No comparator	Participants listened to mindfulness recordings between 8 and 10 min in length whilst wearing virtual reality goggles showing the illusion of floating down a 3D computer-generated river	-Beck Depression Inventory Fast Screen -The Spielberger State-Trait Anxiety Inventory -Graphic Rating Scale (to measure sadness, fear, anger, guilt, shame, disgust, and joy, and acute stress disorder/PTSD)	None.	No statistical analysis run. Participant 1 underwent intervention four times: on day one, depression scores reduced from 8 to 6 out of 10 on a numerical rating scale after intervention. Anxiety reduced from 8 to 6 out of 10 after intervention. Participant 2 underwent intervention twice: on day one, ratings of depression fell from 6 to 5 out of 10 after intervention. Feeling anxious dropped from 7 to 6 out of 10 post-intervention.
Hearn & Finlay (2018)	67	Age (M & SD): Mindfulness group 43.8 (8.7), control group 45.2 (12.2) Gender: Mindfulness group 53% female, control group 55% female Ethnicity: Mindfulness group 78% white British, control group 74% white British Time since injury: Mindfulness group 14% 1–2 years, 62% between 2 and 8 years, 24% 8+ years, control group 19% 1–2 years, 49% between 2 and 8 years, 32% 8+ years Etiology of injury: Mindfulness group 83% traumatic, 17% non-traumatic, control group 66% traumatic, 19% nontraumatic, 16% did not disclose Status: community dwelling	Randomised controlled trial	Experiential avoidance and behavioural flexibility	Intervention: eight-week online mindfulness training course delivered via two ten-minute mindfulness meditations per day, on six of seven days per week. Control: weekly psychoeducational content delivered via email once per week for eight weeks.	Body scanning, breath awareness, mindful movement, acceptance and self-compassion meditations, kindness to others meditations	-Hospital Anxiety and Depression Scale -World Health Organisation Quality of Life BREF -Five Facet Mindfulness Questionnaire -Pain Catastrophising Scale -Pain intensity and unpleasantness (0–10 numerical rating scale)	10 discontinued mindfulness, 5 discontinued psychoeducation. 9 lost to follow-up. Reasons for drop out not reported.	At course completion: significant improvements in depression severity (*p = .*002, partial eta squared (*η*^2^_p_) = .184, 95% CI [− 2.43, − 0.58]), anxiety severity (*p* = .009, *η*^2^_p_ = .137, 95% CI [− 2.60, − 0.40]), pain unpleasantness (*p* = .009, *η*^2^_p_ = .137, 95% CI [− 1.67, − 0.25]), and pain catastrophising (*p* = .02, *η*^2^_p_ = .110, 95% CI [− 4.14, − 0.38]) At three-month follow-up: significant improvements in severity of depression (*p* = .001, *η*^2^_p_ = 0.223, 95% CI [− 3.62, − 1.10]), anxiety (*p* = .023, *η*^2^_p_ = 0.112, 95% CI [− 2.39, − 0.23]), and pain catastrophizing (*p* = .001, *η*^2^_p_ = 0.239, 95% CI [− 5.75, − 1.80]).
Norrbrink Budh et al. (2006)	38	Age (M & SD): Intervention group 53.2 (12.6), control group 49.9 (12.3). Gender: Intervention group 66.7% female, control group 54.4% female Ethnicity: not reported Time since injury: Intervention group 9.9 years (SD 12.8), control group 15.8 years (SD 9.3). Etiology of injury: Intervention group 51.9% traumatic, 48.1% non-traumatic, control group 90.9% traumatic, 9.1% nontraumatic Status: community dwelling	Parallel design	Not reported	Intervention: two sessions per week for a ten-week cognitive behaviour driven programme. Control: no intervention.	Mindfulness and body awareness training	-Pain intensity and unpleasantness (CR10 scale and numerical and verbal rating scale) -Quality of Sleep Questionnaire -Nottingham Health Profile part 1 (QoL) -Hospital Anxiety and Depression Scale -Sense of Coherence Instrument -Use of the healthcare system	None.	No significant change in pain intensity, pain unpleasantness, and quality of life. Anxiety and depression decreased from baseline to the 12-month follow-up. No statistical tests reported.

#### Study appraisal and assessment of risk of bias

The Cochrane Collaboration Risk of Bias Tool, as described in the *Cochrane Handbook for Systematic Reviews of Interventions* [18] was used to evaluate the quality of, and risk of bias in, randomised studies that were included. This assesses risk of bias on a number of domains, such as random sequence generation, and other sources of bias, all rated as ‘high’, ‘low’, or ‘unclear’ risk. High risk of bias in a single domain meant that overall risk of bias was considered to be high. For non-randomised intervention studies, the Effective Public Health Practice Project Quality Assessment Tool (EPHPP [19]) was used. This evaluates internal validity and interpretability of trials on 21 items pertaining to study design, selection bias, blinding, methods of data collection, withdrawals/dropout, and more. Each domain is rated as ‘strong’, ‘moderate’, or ‘weak’. Two independent assessors appraised the studies for risk of bias with good agreement (Cohen’s *k* = .848*, p* < .001).

## Results

### Study selection

**Fig. 1:**
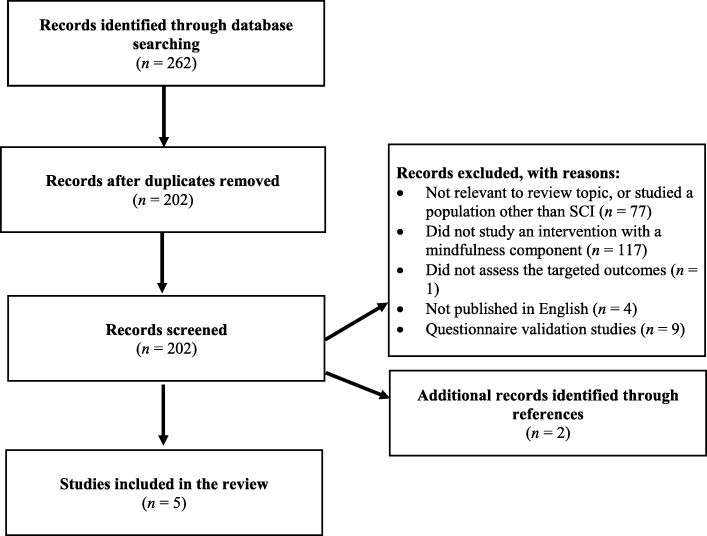
Flow chart search strategy

As of 2nd January 2020, the search protocol yielded 262 articles (50 duplicates subsequently removed). After screening titles and abstracts four remaining studies were deemed relevant for inclusion in the review. However, one study was excluded as the full-text article was unavailable. As a result, a total of three articles were reviewed in full, as well as an additional two, which were identified through screening of reference lists of retained articles (see Fig. 1 for the PRISMA flow chart).

### Sample characteristics

Table 1 presents detailed characteristics of the studies, samples, and findings of studies included in the review. Volunteers willing to take part in the research were sought in all studies, rather than interventions being delivered as standard care. Sample sizes ranged from two to 67, whilst mean age of participants across studies ranged from 31 to 55 years (M = 46.2). Four of the five studies assessed gender [6, 18–20], with three reporting a larger female sample [6, 18, 20]. Time since injury ranged from two weeks to 15.8 years, with largely traumatic injury aetiologies (ranging from 51.9 to 100%).

### Intervention characteristics

Mindfulness training was the central element of the intervention in two studies [20, 21], whilst all others included mindfulness exercises as sub-components of interventions. These focused primarily on six-to-eight weeks of yoga [22, 23] and 2 sessions of CBT per week for 10 weeks [6], with elements of mindfulness training integrated throughout, though these were not described in detail. The CBT programme [6] was delivered in a group format, which included pain education (1.5 h), behavioural therapy (1.5 h), relaxation techniques (1 h), and body awareness training (1 h), a total of 200 h of training. The yoga programmes were delivered in 45–60 min group sessions once per week over the course of eight weeks [22] (a total of up to eight hours of training) and in two 50–60 min classes per week for six weeks [23] (a total of up to 12 h of training), with content including mindfulness meditations, mindful breathing, and seated yoga movements such as neck rolls, with participants instructed to focus on sensations, awareness, and stages of each movement (where participants did not have motor control, they were encouraged to focus on breath awareness or safe modifications were made to accommodate their level of ability). Hearn & Finlay [20] utilised an established online mindfulness training course, which involved participants practicing mindfulness individually (i.e. not in a group format) for ten-minutes, twice per day for eight weeks (totalling 16 h of practice), comparing this in a randomised, controlled design against once-weekly, email delivered psychoeducation on SCI and SCI-specific pain. Another study [21] utilised three short mindfulness practices, each lasting between 8 and 10 min, to enhance virtual reality with two case studies, each of whom took part in the exercises individually (one participant did a mindfulness exercise on four separate occasions, the second participant did mindfulness exercises on two separate occasions). Only one study [22] utilised a mixed-methods approach, and also presented results of qualitative interviews within the paper.

Outcomes targeted by interventions and the measures used can be found in Table 1 (all of which were self-report measures). Whilst all studies examined symptoms of depression, there was a lack of consistency across measures used, with three different questionnaires adopted. Whilst the Hospital Anxiety and Depression Scale was most frequently used (with demonstrated validity in people with SCI [24]), the Beck Depression Inventory has also been found to perform well in SCI populations [25]. However, despite the Positive and Negative Affect Scale being utilised in prior work with people with SCI, this measure has not been validated in this group [26]. Pain-related outcomes were also frequently assessed, with four studies utilising the Brief Pain Inventory [6, 20, 22, 23] (an established reliable and valid measure of pain-related interference in people with SCI and pain [27]). Despite their evidenced role in exacerbating pain and mood problems, only two studies measured pain catastrophising [20, 22] and QoL [6, 20].

### Intervention effects

#### Quantitative results

##### Pain

Of the four studies reporting on pain and pain-related outcomes, only one reported significant improvement in pain unpleasantness and pain catastrophising as a result of intensive mindfulness training immediately post-intervention [20] (pain unpleasantness partial eta squared (*η*^2^_p_) = .137, pain catastrophising *η*^2^_p_ = .110). At three-month follow-up, pain catastrophising continued to improve with a large effect (*η*^2^_p_ = .239). However, this study, and all others measuring pain, reported no change in pain intensity. Three studies [6, 22, 23] reported no change in pain unpleasantness and pain catastrophising post-intervention. Only Hearn & Finlay [20] reported effect sizes in support of the results.

##### Depression

All studies included a measure of depression severity. One study [22] reported no change. All others reported improvements, with two studies reporting significant reductions supported by *p* values [23] and effect sizes and 95% confidence intervals indicating a medium-to-large effect immediately post-intervention (*η*^2^_p_ = .184) and at three-month follow-up [20] (*η*^2^_p_ = .223).

##### Anxiety

Of the four studies measuring anxiety, three of these demonstrated reductions in anxiety [6, 20, 21]. However, only one study [20] supported this with statistical testing (*p* value, effect size, and 95% confidence intervals), which indicated a medium-to-large effect immediately post-intervention (*η*^2^_p_ = .137), and at three-month follow-up (*η*^2^_p_ = .112). One study demonstrated no change [23].

##### Quality of life

Only two studies assessed change in quality of life, despite this being an important goal for people with SCI. Both Norrbrink Budh et al. [6] and Hearn & Finlay [20] reported no significant changes in quality of life.

#### Qualitative results

Only Curtis et al. [22] reported results of qualitative interviews using fundamental qualitative description methodology. The authors asked participants about their expectations of the yoga programme, the aspects they found enjoyable, the things that they did not like or could have been improved, physical and emotional changes seen during and after the programme, and how satisfied they were. Participants reported improved focus, awareness, and ability to be in the present moment as a result of the intervention. They also reported feeling released from day-to-day stress, relaxation, and relief from overwhelming aspects of pain.

#### Adverse events

None of the included studies in this review reported on adverse events arising from participation in the interventions studied.

### Study quality

Weak quality assessments were drawn for the non-randomised studies [6, 21, 22]. These ratings arose due to a range of factors, including low quality study design (e.g. case studies) and small sample sizes (e.g. two participants in one study), lack of control of confounders, and a lack of assessor blinding (see Table 2). Risk of bias across the two randomised studies (Table 3) ranged from high to low, though neither were considered to be completely high quality [20, 23]. For both studies, the extent of reporting bias was difficult to establish. Similarly, the extent of blinding of assessors to outcome measures was unclear, or indicated high risk of bias. Only one study blinded participants to the interventions, thereby posing low risk of bias [20]. Both studies explicitly described how missing data and drop-out was managed, but only one study [20] presented results of between-group statistical comparisons.

**Table 2:** EPHPP Quality assessment for nonrandomised studies included in review

	Quality rating for included studies (strong, moderate, weak)		
EPHPP Risk of Bias Criteria	Curtis et al. [22]	Flores et al. [21]	Norrbrink Budh et al. [6]
Selection bias	Moderate	Weak	Moderate
Study design	Moderate	Weak	Moderate
Confounders	Weak	Weak	Weak
Blinding	Weak	Weak	Weak
Data collection methods	Strong	Weak	Moderate
Withdrawals and drop-outs	Weak	Not applicable	Not applicable
Global Rating	Weak	Weak	Weak

**Table 3:** Cochrane Risk of Bias assessment for Randomised Trials included in review

	Risk of bias rating for included studies (high, low, unclear)	
Cochrane Risk of Bias Criteria	Curtis et al. [23]	Hearn & Finlay [20]
Random sequence generation	Low	Low
Allocation concealment	Low	Unclear
Selective reporting	Unclear	Unclear
Other sources of bias	Low	Low
Blinding (participants and personnel)	Low	Low
Blinding (outcome assessment)	Unclear	High
Incomplete outcome data	Low	Low

Comprehensive sample characteristics, including participant age, gender, ethnicity, time since injury, and injury aetiology, were reported in one study [20] (see Table 1), whilst the other studies omitted important demographic information, such as ethnicity [6, 21, 22], and gender [23]. Further, the sample sizes of the studies were small (average 28). Results reported by Norrbrink Budh et al. [6] and Flores et al. [21] were only described and not reported alongside statistical tests, instead using boxplots and changes in numerical ratings of mood to evidence these changes, respectively. These various limitations may reduce the reliability and generalizability of the findings.

## Discussion

This systematic review aimed to review the efficacy of MBIs for improving pain, depression, anxiety and QoL after SCI, and to make specific recommendations for future research based on the findings. Five studies met the inclusion criteria and were included for review. One study provided the best available evidence of benefits for depression, anxiety, pain-related outcomes and QoL, supported by detailed statistical analyses showing significant results with medium to large effect sizes [20]. All included studies assessed depression as an outcome, thereby highlighting its importance within the population of study, with improvements in both depressive and anxiety symptoms seen across most studies. The findings in this review provide mixed support for the use of mindfulness within interventions to improve outcomes after SCI and highlight the requirement for more rigorous, high-quality research in this area.

The provision of brief (ten minutes) mindfulness exercises via the internet proved a viable and accessible delivery format for an eight-week course [20]. The use of the internet and technology is a factor likely to support people with SCI in more effectively engaging in psychological interventions, given the potential functional, travel, and support-related limitations imposed by the injury [28]. Indeed, studies involving delivery of interventions in a face-to-face format demonstrated conflicting results, which could be an artefact of the potential difficulty faced by people with SCI in attending weekly and twice-weekly sessions over a period of weeks. However, researchers should be explicit in how they track engagement in psychological interventions, given the high drop-out rates noted in some studies. Such non-adherence may arise from the requirement of active, self-motivated participation, which may act as a barrier to engagement [29]. Furthermore, regular follow-up would be particularly useful to assess long-term effects of MBIs after participants have completed an intervention, and will also serve to highlight whether further ‘booster’ sessions are required to maintain benefits.

It is also unclear in the studies the extent to which mindfulness exercises utilised in the courses were tailored/adapted to accommodate the specific physical and sensory function of people with SCI. The use of generic mindfulness exercises, such as the standard ‘body scan’ meditation, may not be wholly appropriate for people with reduced sensory function as a result of neurological injury, which could hinder their opportunities to engage and ability to develop mindfulness skills and experience positive change. It may, therefore, be appropriate for future work to consider the extent to which mindfulness interventions need to be modified to accommodate reduced sensory function and the physical accessibility of face-to-face courses.

A major issue with the studies included in this review is that four included mindfulness training/meditations as a secondary component to interventions that were focused primarily on either yoga, virtual reality, or CBT, making it difficult to establish the extent that improvements reported are attributable to improvements in mindfulness or other elements of the interventions. This finding reflects previous systematic review work examining the efficacy of MBIs for people with multiple sclerosis [30], demonstrating the broader need for work to establish the mechanisms of change in studies including mindfulness components. Only one study evaluated the efficacy of an intervention that was solely focused on mindfulness training [20]. This study evidenced benefits to depression, anxiety, pain-related outcomes and QoL, and reported comprehensive statistical tests, thereby providing the best currently available evidence for the efficacy of mindfulness for secondary consequences of SCI.

Whilst the efficacy of mindfulness skills training for people with SCI remains under-studied, the current review suggests that integrating mindfulness components into interventions for people with SCI may hold promise for some aspects of life after SCI. Unfortunately, however, there still exist a number of gaps in the literature that should be explored, such as the long-term benefit and efficacy of brief, intensive MBIs, as well as their cost-effectiveness and the optimal ‘dosage’/frequency of practices. A key advantage of the current review is the ability to make recommendations for future, high-quality work, such as adopting rigorous protocols, recruiting larger samples, and recommending the examination of the comparative efficacy of MBIs through the use of active interventions (vs. waitlist control groups). Similarly, exploring the impact of mindfulness on people with SCI through the use of *N*-of-1 studies is recommended to further highlight the unique, individual-level benefits and, importantly, any potential adverse consequences of mindfulness practice in this population.

### Limitations

Despite the rigorous search criteria adopted, and comprehensive reporting of the review, there are limitations of the review to consider. First, there were major methodological differences between the studies reviewed, which limit the generalisability of the results. For example, there was a lack of consistency in outcome measures; four different measures were used to assess depression and anxiety, each of which may define each outcome in slightly different ways. Similarly, sample sizes were relatively small, though it is acknowledged that recruitment of samples with SCI can be challenging due to the small populations. Two studies failed to provide statistical results in support of their findings; only one study provided statistical measures of change, including *p* values, effect sizes, and 95% confidence intervals. Future work could consider using within-person units of analyses such as Ecological Momentary Analysis [31] or N-of-1 RCTs [32, 33] for tailored interventions.

## Conclusions

This review highlights areas of priority for future research into MBIs for people with SCI. There exists only one randomised controlled trial published in this area, which was the only study to utilise a programme solely dedicated to improving mindfulness skills, thereby demonstrating a clear need for more high-quality trials, particularly with long-term follow-up, to establish more definitive conclusions. The small number of studies included in the present review mean that conclusions drawn are preliminary and thus reflects the paucity of the research in the area to date.

## Data Availability

All data generated or analysed during this study are included in this published article.

## Abbreviations

CBT: cognitive behaviour therapy
EPHPP: `Effective Public Health Practice Project
MBI: mindfulness-based intervention
PRISMA: Preferred Reporting Items for Systematic Reviews and Meta-Analyses
QoL: quality of life
RCT: randomised controlled trial
SCI: spinal cord injury
TENS: transcutaneous electrical nerve stimulation
